# The Influence of Talc Addition on the Performance of Polypropylene Membranes Formed by TIPS Method

**DOI:** 10.3390/membranes9050063

**Published:** 2019-05-14

**Authors:** Marek Gryta

**Affiliations:** Faculty of Chemical Technology and Engineering, West Pomeranian University of Technology Szczecin, ul. Pułaskiego 10, 70-322 Szczecin, Poland; marek.gryta@zut.edu.pl

**Keywords:** membrane distillation, membrane stability, polypropylene, TIPS, talc

## Abstract

The effect of talc addition on the morphology of capillary membranes formed by a thermally induced phase separation (TIPS) method was investigated in the presented work. The usability of such formed membranes for membrane distillation was evaluated. Two types of commercial capillary polypropylene membranes, fabricated for microfiltration process, were applied in the studies. A linear arrangement of polymer chains was obtained in the walls of membranes formed without a talc addition. In the case of membranes blended with talc, the linear structure was disordered, and a more porous structure was obtained. The changes in morphology enhanced the mechanical properties of blended membranes, and their lower thermal degradation was observed during 350 h of membrane distillation studies. Long-term studies confirmed the stability of talc dispersion in the membrane matrix. A leaching of talc from polypropylene (PP) membranes was not found during the membrane distillation (MD) process.

## 1. Introduction

The appearance of the MD process can be found in the Bodell patent from 1963 [1,2] and despite the intensive studies carried out for more than 50 years, durable MD membranes have not yet been developed. The hydrophobic porous membranes made from polytetrafluoroethylene (PTFE), polyethylene (PE), polyvinylidene fluoride (PVDF) and polypropylene (PP) are frequently used in the studies of the MD process [2,3,4,5,6,7]. In several works, the commercial membranes made from these polymers for microfiltration (MF) were tested as the MD membranes [3,8,9,10,11,12]. However, with regard to the MD process conditions (e.g., higher feed temperature and non-wetted pores) the membranes fabricated for MF, in the majority of cases, did not fulfil the MD process requirements. Generally, a membrane for the MD process has to simultaneously meet the following requirements [2,12,13,14,15,16,17,18,19]:

- high liquid entry pressure (LEP), which is the minimum transmembrane hydrostatic pressure that is applied on the membrane before a liquid solution penetrates into the pores;
- good thermal stability—up to the boiling temperature of water;
- high chemical resistance to separated solutions;
- high permeability;
- low thermal conductivity;
- narrow pore size distribution.

A simultaneous fulfilment of these conditions is difficult, e.g., high permeate flux can be achieved for thin membranes, but a low thermal conductivity and higher energy efficiency can be obtained when thicker membranes are used, especially for brines desalination [20,21]. Moreover, a further progress of the MD process (industrial implementation) requires a substantial growth in studies on a semi-pilot or pilot scale [18,21,22].

The pioneering activity within a scope of the MD process implementation is performed, among others, by SolarSpring GmbH (Freiburg, Germany), Memsys GmbH (Schwabmünchen, Germany), TNO (Den Haag, The Netherlands), Aquaver (Voorburg, The Netherlands), Scarab Development AB (Stockholm, Sweden), Xzero AB (Stockholm, Sweden), BlueGold Technologies (Largo, FL, USA) and Abengoa Water (Seville, Spain) [18]. The pilot plants constructed by the above-mentioned companies utilize mainly the membranes made from PTFE, PE and PP recently produced for the MF process [18,23]. Although the applied membranes exhibit certain drawbacks, the realized pilot studies have a positive impact on the demand for the MD process and enhance the opportunity for implementation of novel membrane production in future.

An intensive development of the MD process investigation has been observed in recent years, and as a result, the number of publications has grown from 1000 to almost 5000 [16,18]. Several works have reported that the pores wettability can be restricted by application of composite membranes or membranes with modified surface which enhances the resistance to wetting [8,9,16,24,25,26]. Promising results were obtained for the membranes formed by electrospinning method and the membranes with addition of different fillers, e.g., carbon nanotubes, titanium and silicon dioxide or talc [27,28,29,30,31,32]. In addition to membranes made of polymers, ceramic membranes are also presented, which create a chance for applications requiring particularly durable membranes [33,34]. Many new types of membranes and methods for their preparation and the possibility of their use in the MD process are described extensively in review articles [8,16,18,34,35].

Nevertheless, so far, no breakthrough results have been obtained, which indicates that durable membranes for the MD process could be difficult to fabricate. One of a reasons for the slow development of MD membranes is the fact that the majority of works present only ways to increase the LEP value or contact angle, but there are no studies demonstrating the durability of new types of membranes during a long-term module exploitation. Moreover, even the membranes with a very good resistance to wetting, can undergo the progressive degradation due to slow changes in the polymer after 100–200 h of the MD process [36]. Moreover, the implementation of new types of membranes for the production is difficult, mainly due to a fact that the MD membranes market is just being created [18]. Moreover, the manufacture of MD membranes on a larger scale is necessary because it enables the performing of pilot studies [18,22]. Demonstrating the possibility of realizing different applications of the MD process on a pilot scale gives the opportunity to attract industrial investors, what is necessary for process implementation.

With regard to the above-mentioned issues, at the initial stage of MD development it is essential to apply the methods already industrially employed for the production of MD membranes. A thermally induced phase separation (TIPS) process is such a method, commonly used to fabricate the membranes from hydrophobic polymers [1,37,38]. In the TIPS method, the polymer with/without fillers is introduced into the mixer, where is mixed with proper amounts of different kinds of oils [37,38,39,40,41,42,43]. The polymer granulates are completely melted in the diluents system, and obtain a hot (e.g., 453–493 K) homogenous dope solution that flows through the spinning nozzle into a coagulation bath. Subsequently, the oils are extracted from the membrane matrix.

It has been demonstrated in numerous papers, that the membranes manufactured by the TIPS method have the appropriate properties for the MD process. The membranes made from PP are most often used for MD [10,18,23,37,38,41,43]. Moreover, the application of other polymers, such as PVDF, PE and polyethylene chlorinetrifluoroethylene (ECTFE) for MD membrane preparation via the TIPS method is also possible [39,44,45]. In several works it has been reported that improvement of the properties of MD membranes could be obtained by modification a composition of dope solution and by using different conditions for the membrane formation. However, in the majority of cases, the studied membranes were formed under laboratory conditions. Therefore, certain phenomena taking place during the membranes production on the industrial scale, such as the row arrangement of polymer chains resulting in the formation of linear structure of the membrane matrix, can affect the polymer mechanical properties [46]. The possibilities of preventing the polymer degradation and methods for improvement of the mechanical properties of PP membranes by introducing fillers into the dope solution, are presented in this paper.

The polypropylene capillary membranes represent the membranes manufactured via the TIPS method on an industrial scale for the MF process [10,23]. The flow of melted polypropylene through a spinning nozzle causes a more linear arrangement of the polymer chains. A cooling-down of formed capillary in the coagulation bath proceeds rapidly [38], which favours the freezing of polymer linear structure. A result is a deterioration of tensile strength manifested by a longitudinal creaking of polymer. The addition of crystals nucleus (e.g., inorganic fillers) into a dope solution is one of the methods of disturbance of the linear arrangement of polymer chains [46,47]. It is well-documented in the literature that the blending of polymers with mineral fillers is considered a useful way to improve the mechanical properties and talc is a popular mineral filler used for this purpose [46]. Talc is a cheap filler and more importantly, it has the hydrophobic properties that enable its very good dispersion in polyolefin. For this reason, talc is often used to improve the properties of materials produced from polyethylene or polypropylene [30,47].

In current MD pilot studies, hydrophobic membranes produced for microfiltration are often used. However, the feed temperature in the MD process, as a rule, is higher than that which is applied in the MF process. As a result, the membrane matrix is subjected to a larger thermal expansion during the MD process, what can lead to damages in the membrane structure. In order to enhance the thermal resistance of produced polymeric materials from PP, the polymer is usually blended with different inorganic fillers, such as talc, which exhibits many actions improving the PP properties [29,47]. It was demonstrated that the application of talc enhanced the thermal resistance and tensile strength of PP membranes [29,39,48,49]. However, as the thickness of the film produced from PP decreases, then the beneficial effects of the fillers used, such as talc, are reduced [46]. Moreover, the structure of the membranes (pore walls) has a much smaller thickness compared to the films produced. For this reason, the objective of this work is to determine the effect of talc addition on the properties of PP membranes used in the MD process. Moreover, PP membranes have been shown to undergo a significant transformation, such as surface wettability, during initial 50–100 h of the MD process [10,36,50]. Therefore, the possibility of application of used membranes in this work over significantly longer periods (350 h) was studied.

The membrane matrix durability studies were often omitted in the previous works describing the MD process. One of the main applications of the MD process is water desalination in order to obtain drinking water. Works in which research is undertaken to determining whether the fillers introduced into the membrane matrix or compounds used to modify the membranes surface will not be released into the produced water are scarce [51]. This information is important since many of the components, such as nanomaterials, used to improve the properties of MD membranes, are suspected of being carcinogenic [52,53]. There are well-known works suggesting that a long-term contact with talc may also have a carcinogenic effect [54]. Until now, such an action has been attributed to talcum forming asbestos-moulded structures [54,55]. However, recent studies indicate that the so-far considered safe platelet forms of talc may also have a negative effect on human health [56]. For this reason, it is important to obtain a kind of membrane matrix that would prevent the leaching of fillers. The present study investigated whether there was no leaching of talc from the surface of the membranes during a long-term MD process.

Taking into account a significant impact of production installation design and the conditions of its exploitation on the properties of fabricated membranes, the studies were performed using two types of capillary MF membranes (with/without talc) manufactured in industrial installation under similar conditions of the TIPS process.

## 2. Materials and Methods

Two types of capillary PP membranes purchased from PolyMem (Warszawa, Poland) were used in the studies. These membranes have the internal diameter of 1.8 mm and the outer diameter of 2.6 mm. Although the composition of dope solution and the process parameters of membrane production were similar, about 10% of talc (as a nucleation agents) was added to one dope solution. The membranes utilized in this work were designated as PP-N (net polypropylene) and PP-T (polypropylene with talc). The parameters of used membranes were summarized in Table 1. The manufacturer stated that the average pore diameters equal to 0.2 μm were represented in both cases. The membrane porosity was determined using the gravimetric method [57]. In this case, the membrane samples with a length of 25 cm were soaked in isopropanol for 1 h to achieve a pore wetting.

**Figure 1:**
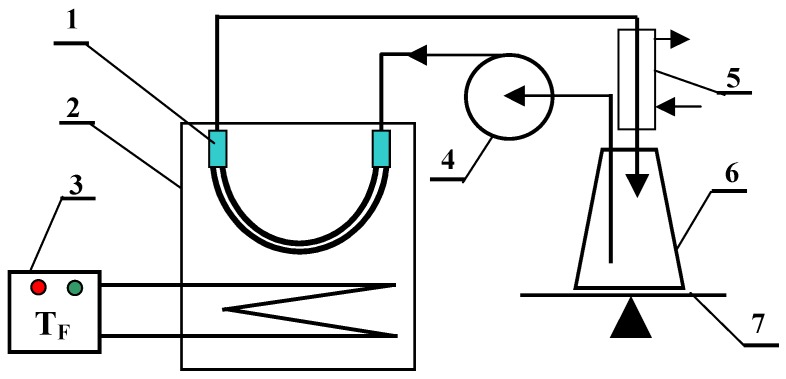
MD experimental set-up. 1—MD module, 2—feed tank, 3—Nűga temperature regulator, 4—peristaltic pump, 5—cooler, 6—distillate tank, 7—balance.

The MD module configuration, such as submerged modules, was applied for direct contact MD studies with preferred option. Each module was equipped with two capillaries, and the working length of capillaries was 40 cm (membrane area 45 cm^2^). The experimental set-up is shown in Figure 1. The submerged MD module was assembled inside the feed tank, and distillate flowed inside the capillaries (linear velocity of 0.55 m/s). The MD installation was operated, at the feed temperature equal to 353 K, for 350 h in a continuous mode (day and night). Since mainly the thermal effects were studied, a dilute NaCl solution (1 g/L) was used as a feed. When certain pores are wetted in the membrane, the feed may leak to the distillate. Therefore, the presence of NaCl increases the electrical conductivity of the distillate, which allowed for assessing the degree of wetting of tested membranes. The distillate was cooled by tap water and its temperature was maintained at 288–295 K. The permeate flux was calculated on a basis of changes in the distillate volume over a studied period of time (20–24 h). The volume of obtained distillate was in the range of 500–1000 mL over this period. Assuming that the volume was measured with an accuracy of 5 mL and the error of the membrane area calculation was 2%, the error of the MD measurements (permeate flux) did not exceed 2–3%.

The thermal properties of used PP membranes were determined by differential scanning calorimetry (DSC). These tests were performed by means of a device DSC Q100 (TA Instruments, New Castle, DE, USA), at heating and cooling rate of 10 K/min within the temperature range 200–523 K. The samples were examined in heating-cooling-heating cycles after previous drying.

The mechanical strength and elongation at break of the PP capillary membrane were measured with a tensile tester (3366 Universal Material Testing Machine, Instron, Norwood, MA, USA) according to PN-EN ISO 527-1:1998 method. The specimens of membrane with an initial length of 10 cm were clamped at both ends obtaining the measurement length 3 cm, and pulled in tension at the constant elongation rate of 10 mm/min. In each case, the measurements were performed for ten samples of PP membrane.

The changes of membrane hydrophobicity were determined using a Sigma 701 microbalance (KSV Instrument, Ltd., Espoo, Finland). Based on the Wilhelmy plate method, the dynamic contact angles were measured.

A 6P Ultrameter (Myron L Company, Carlsbad, CA, USA) was applied to measure the values of the electrical conductivity.

The membrane morphology and composition of inorganic fillers were investigated using a Hitachi SU8000 Scanning Electron Microscope (SEM) equipped with Energy-dispersive X-ray Spectrometer (EDS). The specimens for cross-sectional examinations were prepared by fracture of the capillary membranes in liquid nitrogen. Before the SEM examination the membrane samples were sputter coated with chromium (about 3–5 nm) using Q150T ES coater (Quorum Technologies Ltd., Lewes, UK).

In addition to SEM-EDS examinations, the membranes were tested using FTIR, XPS and elemental analysis. This analysis was performed using Elemental Analyzer FLASH 2000 CHNS/O (Thermo Scientific, Waltham, MA, USA), which operates according to the dynamic flash combustion of the sample. For C and H determination the obtained gases were carried by a helium flow to a layer filled with copper, then swept through a GC column, and finally, a Thermal Conductivity Detector (TCD) detected them. In the case of oxygen, the samples were introduced into the pyrolysis chamber via the MAS Plus Autosampler. The reactor contains nickel-coated carbon maintained at 1060 °C. The oxygen in the sample forms carbon monoxide, which is then separated from other products using gas chromatograph and detected by the TCD Detector.

The functional groups presented on the membrane surface were identified by a Fourier transform infrared (FTIR) spectroscopy. The method of Attenuated Total Reflection (ATR) was applied. In the performed studies, a Nicolet 380 FTIR spectrofotometer connected with Smart Orbit diamond ATR instrument (Thermo Electron Corp., Waltham, MA, USA) was used.

Chemical composition of the membrane surfaces was evaluated by X-ray photoelectron spectroscopy (XPS) (Prevac, Rogów, Poland). Prevac electron spectrometer, equipped with an SES 2002 (VG Scienta, Uppsala, Sweden) electron energy analyzer working in a Constant Energy Aperture mode was applied in these studies. The concentration of detected elements (expressed in atomic percents) was calculated in CasaXPS software.

X-ray diffraction (XRD) studies were performed in order to determine a crystal structure of the membranes. In these studies was applied an EMPYREAN diffractometer (PANanalytical, Almelo, The Nederland) using a monochromatized CuKα radiation (35 kV, 30 mA). The obtained peaks that presented the kth Bragg reflection were described by Pseudo-Voigt profile function, which can be assigned a fixed shape of any type between their limiting Gaussian and Lorentzian forms. Based on the Pseudo-Voigt profile, the High Score Plus 3.0 software was applied for estimation of a full-width at half maximum (FWUH) parameter. XRD allowed for indicating a material with different electron density. Assuming an existence of a difference between PP and talc, a trial was undertaken to apply software Easy SAXS 2.0 for determination of talc particle size.

## 3. Results and Discussion

### 3.1. SEM Examinations of Membranes

**Figure 2:**
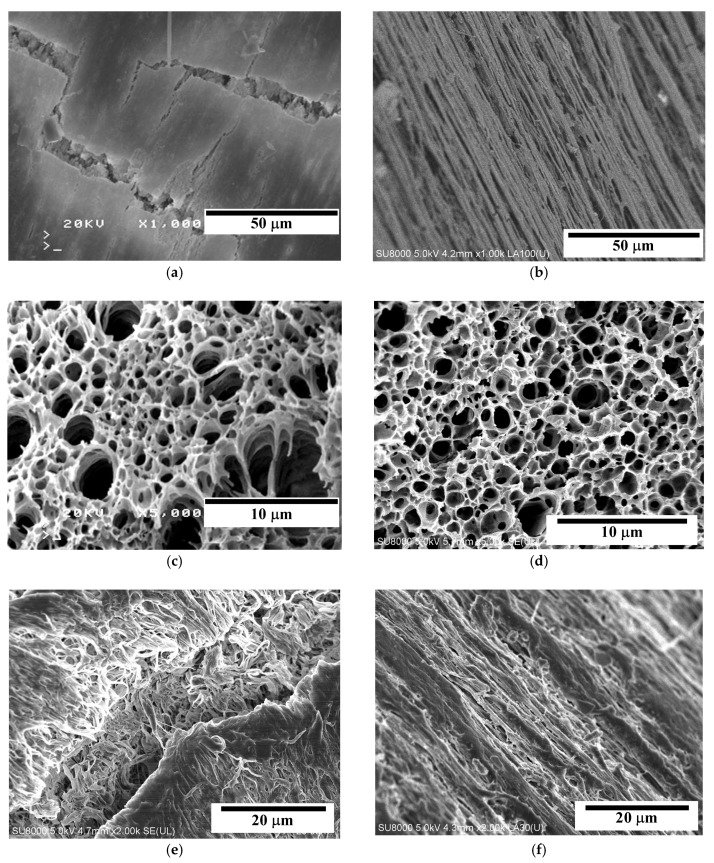
SEM images of new capillary membranes. Membrane PP-N: (**a**) external surface, (**b**) internal surface, (**c**) cross-section. Membrane PP-T (with talc): (**d**) cross-section, (**e**) external surface, (**f**) internal surface.

The performed SEM examinations revealed significant differences in the structure of tested membranes, especially that the surfaces of PP-T membranes blended with talc were more porous (Figure 2). These results confirmed that the addition of fillers into a dope solution significantly influences the morphology of membranes formed by the TIPS method [41,42].

In each case studied it was found that both the internal and external surfaces of the capillaries were less porous than a structure formed inside the membrane wall (Figure 2). A reason for different porosity is associated with the leaching of solvent and the rate of cooling, which takes place at a significantly faster rate on the capillary surface than inside the wall [45]. Moreover, in the case of PP-N membrane (without talc) the porosity of the external surface of capillary (Figure 2a) was lower than that observed on the lumen side (Figure 2b). The characteristic row lamellar structure was formed on this side. When a dope solution flowed through the spinning nozzle, the higher shear rate provided the necessary conditions to form highly oriented row nuclei, which induce an epitaxial growth of crystallites [46]. As a result, a linear layer of PP chains oriented parallel to the flow direction of dope solution was formed.

In the case of PP-T membranes blended with talc, the linear structure was disordered, and a more porous surface was obtained (Figure 2e,f). The final morphology depends on the relaxation behavior of the PP chains during the cooling and solidification process of casting dope [46]. Long polymer chains with a longer relaxation time did not have sufficient time to relax and would remain in the stretched state. The talc addition increases the number of nuclei; therefore, the crystallization rate of PP is significantly accelerated [29,39]. This meant that long polymer chains (linear structure) were not formed or/and that the relaxation time was shorter, and that the PP chains were able to quickly relax back to the coiled state.

Inside the membrane wall the cooling and solidification processes proceed at a significantly slower rate, thus, the polymer chains have sufficient time to relax, and the porous structure without linear orientation can be formed [46]. The SEM examinations revealed that a sponge-like structure was formed in both cases (Figure 2c,d). The addition of talc meant that the pores in the PP-T membrane were more spherical and the smaller pores with the dimension of pore cells in the range of 1–2 μm were formed. Definitely larger differences in the pore size were exhibited in the PP-N membrane. Besides the pores with dimension of 1–2 μm, numerous pores with the size of 3–7 μm also occurred (Figure 2c). Such large pores may facilitate the wetting of PP-N membranes during the MD process.

**Figure 3:**
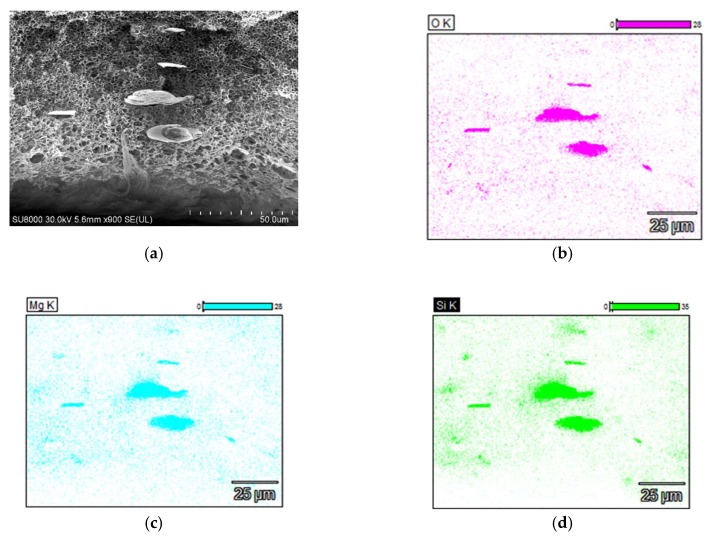
SEM-EDS analysis of PP-T membrane cross-section (**a**). Elements dispersion: (**b**) oxygen, (**c**) magnesium, (**d**) silicon.

The SEM examinations of PP-T membrane did not reveal the presence of larger talc particles (i.e., size above 0.1 μm) in the membrane matrix and on the capillary surfaces (Figure 2c–f). The presence of talc particles with a significant size (e.g., 5–10 μm) was only found in a few places of the examined cross-section (Figure 3a). However, the SEM-EDS examinations showing a good dispersion of small talc particles (chemical formula Mg_3_Si_4_O_10_(OH)_2_) in the membrane matrix (Figure 3b–d). This indicated that a dominant fraction of added filler (talc) had a smaller size than that which was observed in Figure 3a.

**Figure 4:**
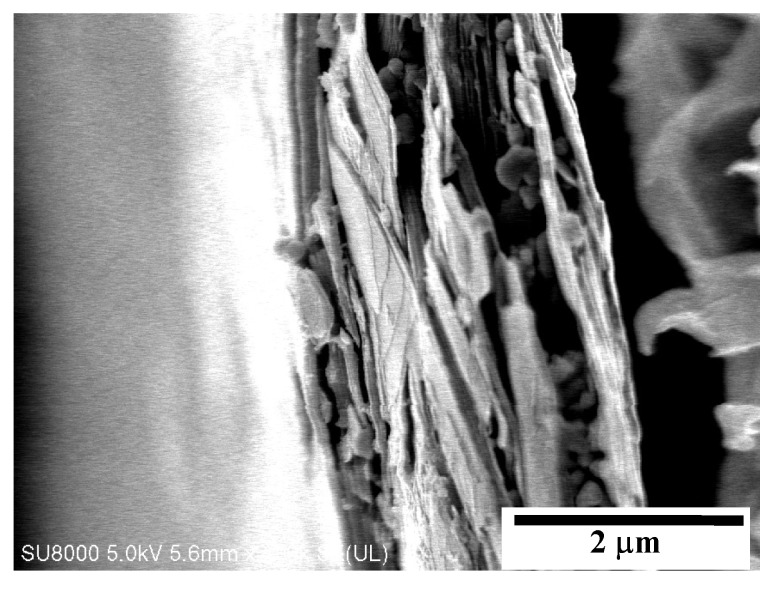
SEM image of talc particle observed inside the PP-T membrane cross-section.

The SEM observations revealed that the talc used for membranes production had a plate structure (Figure 4). The point analysis of the composition made by the SEM-EDS method showed that it contained 46.1% O, 13.1% Mg, 16.4% Si and 24.4% C. Carbon is not a component of talc, but in the case of particles of size about 2 μm (Figure 4) the EDS analysis also includes polypropylene surrounding such a small particle.

**Figure 5:**
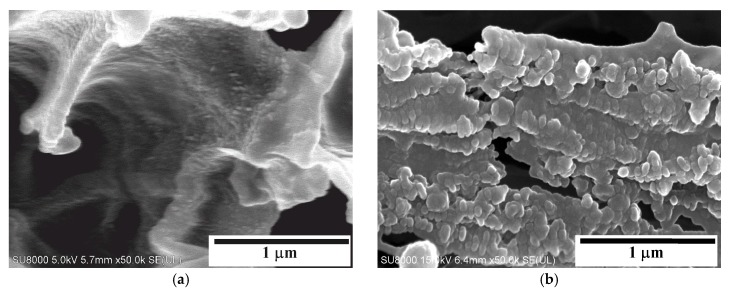
SEM image of the PP-T membrane cross-section. Magnification 50,000×. (**a**) surface inside the pore; (**b**) shish-kebab crystal structures.

The SEM observations of PP-T membrane samples conducted under high magnification (50–100 k) enabled us to conclude that there was no talc particle size above 50 nm on the surface of the membrane matrix (Figure 5). On the other hand, such large magnifications allowed for observing the formation of shish-kebab crystal structures (Figure 5b), which is characteristic of polypropylene and which can be prepared in polymeric materials by adding nanoparticles or fibres [58]. Such forms were not found when observing PP-N membrane samples. This confirms the beneficial effect of talc as nuclei accelerating the PP crystallizations.

An attempt was made to determine the size of talc particles based on XRD measurements. A particle size analysis conducted with the use of Easy SAXS software showed most frequent radius equal to 68.9 nm, and the obtained value of relative standard deviation was 44.4%. Although this result does not contradict the conclusions from SEM observations, it can be burdened with a large method error. The main reason for this is a very low absorption factor of sample (1.313), which may indicate that PP is too light a material (low electron density) for this method. Moreover, the membrane matrix also contained talc particles with sizes over 1000 nm, which are too large for this method and thus could distort the measurement results.

### 3.2. Long-Term MD Studies

The MD process was carried out using the submerged modules located in the feed tank, and the distillate flows on the lumen side. In the performed MD studies, the feed temperature amounted to 353 K. The application of such temperature or higher, allows for significantly increasing the efficiency of the MD process [10], but disadvantageously accelerates the polymer degradation process. In our case, a high feed temperature applied in the studies carried out for 350 h should facilitate the evaluation of effectiveness of talc addition to the MD membranes. The membrane degradation causes the degree of PP surface oxidation (hydrophilization) to increase and the formation of cracks in the capillary wall can be observed [46]. The hydrophilization of membranes causes a systematic decline of the module productivity and the values of electrical conductivity of obtained distillate are increased [2,9,16,22].

**Figure 6:**
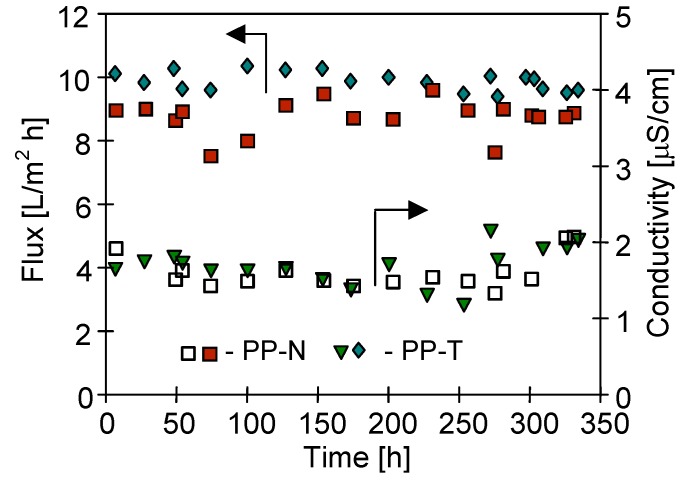
The changes of permeate flux and distillate conductivity during MD process. T_F_ = 353 K.

The changes of the permeate flux and electrical conductivity of distillate during long-term MD studies are presented in Figure 6. A comparison of experimental results indicated that the obtained permeate flux for each tested membrane was practically constant during the MD study period (350 h). Small flux discrepancies resulted from the changes in cooling water temperature (Figure 1), because for reasons of safety, the distillate side thermostat was abandoned (additional electric heater). The largest productivity, at a level of 10.4 L/m^2^h was obtained for the PP-T membrane and a slightly smaller result (9.5 L/m^2^h) for PP-N membrane. The observed stability of the permeate flux confirms that the process of membrane wettability is restricted.

A higher permeate flux obtained for PP-T membranes results from a larger porosity of these membranes especially for the external surface of capillaries, as was demonstrated by the SEM examinations (Figure 2). These results are also in agreement with those obtained in the previous work, in which the permeate flux was lower by more than 10%, due to the decrease in the surface porosity of used membranes (as in the case of PP-N) [59]. Such a small decrease in efficiency was also obtained in other works wherein the strength of MD membranes was increased by applying an additional surface layer. Such a layer allows for significantly increasing the value of LEP and the contact angle of the membrane surface [24,25,35]. However, this method also increases the surfaces for heterogeneous crystallization. Therefore, to assess the effectiveness of using the membranes for pilot-plant studies, long-term MD studies with intensive scaling should be carried-out.

The addition of inorganic fillers can accelerate the membrane wetting [60]; therefore, a growth of the electrical conductivity of the MD distillate can be observed. However, the talc is regarded as a hydrophobic material [29,30], thus, it should not facilitate the penetration of water into the pores. This conclusion was supported by a low value of the electrical conductivity of the obtained distillate. The conductivity values at a level of 2 μS/cm were stable for distillate obtained from each module (Figure 6). Taking into account the conductivity of the feed exceeding 2000 μS/cm, it can be concluded that the degree of separation obtained for each of the tested membranes was close to 100%. Such a result and the stable permeate flux confirms that the used PP membranes exhibited a good resistance for wettability.

**Figure 7:**
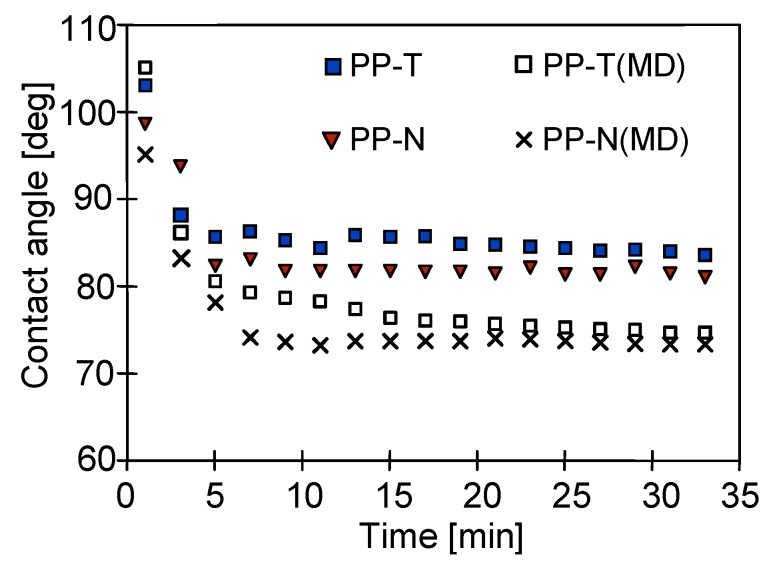
The changes of the contact angle (advancing) during continuous measurements of dynamic contact angle using the Wilhelmy plate method. Samples of new membranes and membranes collected from modules after 350 h of MD process.

The membrane wettability is characterized by a value of the contact angle. The dynamic measurement of the contact angle was carried out using the Wilhelmy plate method. The initial values obtained for new membranes were 99° and 104° for PP-N and PP-T membranes, respectively. A morphology of the membrane surface is strongly affected by the value of the contact angle during the first immersion of membrane samples, however, after 20–30 repetitions of immersion–emersion cycles the contact angle values were stabilized, and the values at a level of 82° (PP-N) and 86° (PP-T) were obtained (Figure 7). Similar values of the contact angle were obtained: for PP-N 73.2° and 74.6° for PP-T membranes collected from modules after 350 h of MD process; it can thus be concluded that the addition of talc increased, in a slight degree, the resistance of tested PP-T membranes to wetting. This was mainly due to the fact that the addition of talc to a dope solution allowed for obtaining the pores of similar sizes and eliminating the formation of very large pores, which are easier to wet.

**Figure 8:**
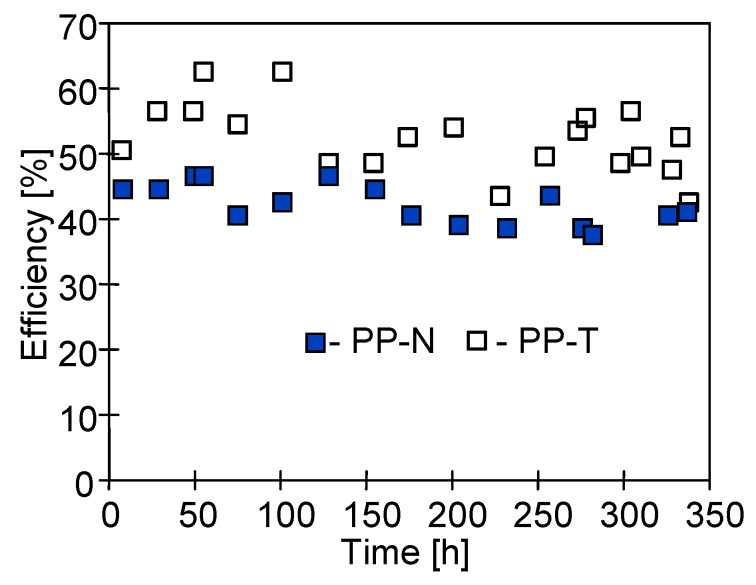
The changes of thermal efficiency of used membrane modules during the MD process.

The addition of inorganic fillers into the polymeric membrane matrix can enhance the values of thermal conductivity and as a result; the heat losses will be higher in the MD process. However, the results presented in Figure 8 indicate that a higher thermal efficiency (55%) was obtained for the PP-T membrane in a comparison with the PP-N membranes without the talc additive (efficiency 43%). It can be concluded that for tested membranes, the magnitude of obtained permeate flux mainly determines the thermal efficiency of the MD process.

### 3.3. Membrane Performance

A polymer with the linear structure formed on the surface of PP-N membranes can reduce the tensile strength of the membrane matrix. As a result, multiple cracks, probably formed during the membrane production, were observed on the external surface (Figure 2a). A long-term annealing of PP enables the rearrangement of PP chains in the membrane matrix, which improves the lamellae orientation and uniformity [61]; hence, further damages/cracks can be expected during the MD process.

**Figure 9:**
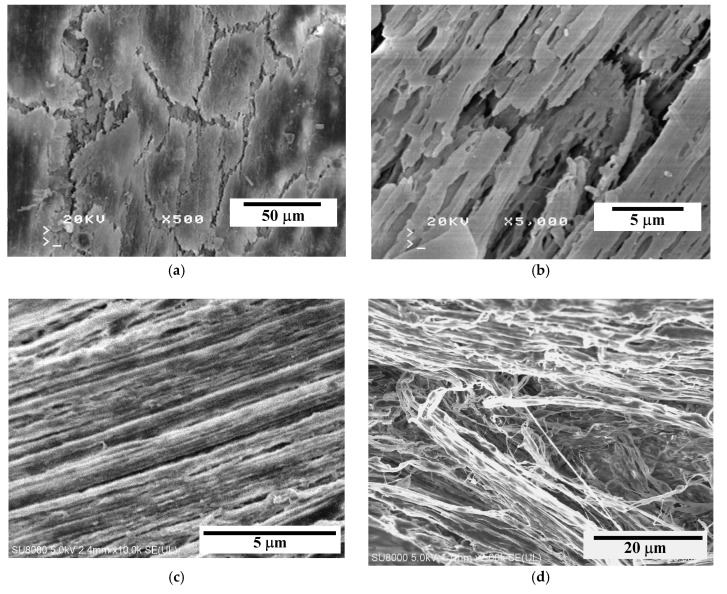
SEM images of PP membranes after MD process. Membrane PP-N: (**a**) external surface; (**b**) external surface magnification; (**c**) internal surface; (**d**) external surface of PP-T membrane.

The performed SEM examinations of membrane samples collected from MD modules after 350 h of their exploitation confirmed that a considerable degradation took place in the membranes without the talc addition. The external surface of PP-N membrane with visible numerous cracks is shown in Figure 9a. A number and the dimensions of these cracks are definitely larger in comparison to those observed on the surface of new membranes (Figure 2a). An enlarged image of cracks (Figure 9b) shows that the linear structures of PP chains undergo, not only a breakage but also a split of the membrane surface along the formed structures. These kinds of damages on the lumen side of PP-N membrane were found to occur to a lesser degree (Figure 9c). However, on this side the distillate flows at a low temperature (below 313 K). This confirms an assumption that a high feed temperature is the main reason for a stress that resulted in damage of the surface structure in the tested PP-N membranes. A phenomenon of thermal extension generates the stress also inside the PP-T membrane. However, talc addition improved the cellular structure, which is more resistant on the stress than a linear structure, and thus, the degradation of the membrane matrix was not observed (Figure 9d). The SEM examinations of PP-N membrane cross-sections revealed that a lack of cracks inside the capillary wall was due to the same reason. A fact, that the degradation of PP-N membrane structure was limited to its external surfaces was also confirmed by the results of the MD process, in particular, by low values of electric conductivity of the obtained distillate (Figure 4).

Based on the above results, it can be concluded that a cellular structure occurring inside the capillary wall is definitely more resistant to the stress formed in the membrane in comparison with a linear structure of polymer formed on the membrane surfaces. This conclusion was confirmed by the results of tensile tests (Table 2). The values of all the studied parameters (Young’s modulus, elongation at break and tensile strength) were better for membranes blended with talc, having a mainly sponge-like structure in comparison to that observed for PP-N membranes.

The mechanical strength of MD membranes is as important as their resistance to wetting. Even small cracks can cause a significant leakage of the feed. In the case of PP membranes, their strength depends, to a large extent on the degree of crystallinity and the structures that constitute the polymer chains.

The addition of talc accelerates the formation of crystallites and decreases the spherulite dimensions, thus improves the thermal and mechanical properties of polymer matrix [39,49]. The results of DSC analysis (Table 3) indicated the increase of melting enthalpy (crystallinity-X) and the crystallization temperature, which confirmed that talc is a good nucleating agent. However, the degradation processes occur during MD module exploitation, which would result in a remarkable deterioration in the membranes properties. The high feed temperature applied in the MD process can accelerate the thermal degradation of polypropylene [36,50]. The degree of degradation of the PP membrane matrix was determined in the DSC studies. In each case, it was observed that the membranes after the MD process exhibited a reduction of T_m_ values, which was larger (from 164.6 to 157.2 °C) in the case of PP-N membranes (Table 3). It has been reported that the thermodynamic melting temperature of semicrystalline polymers decreases as the molecular weight decreases and/or as the number of defects increases with an increase in branching and/or crosslinking [51].

**Figure 10:**
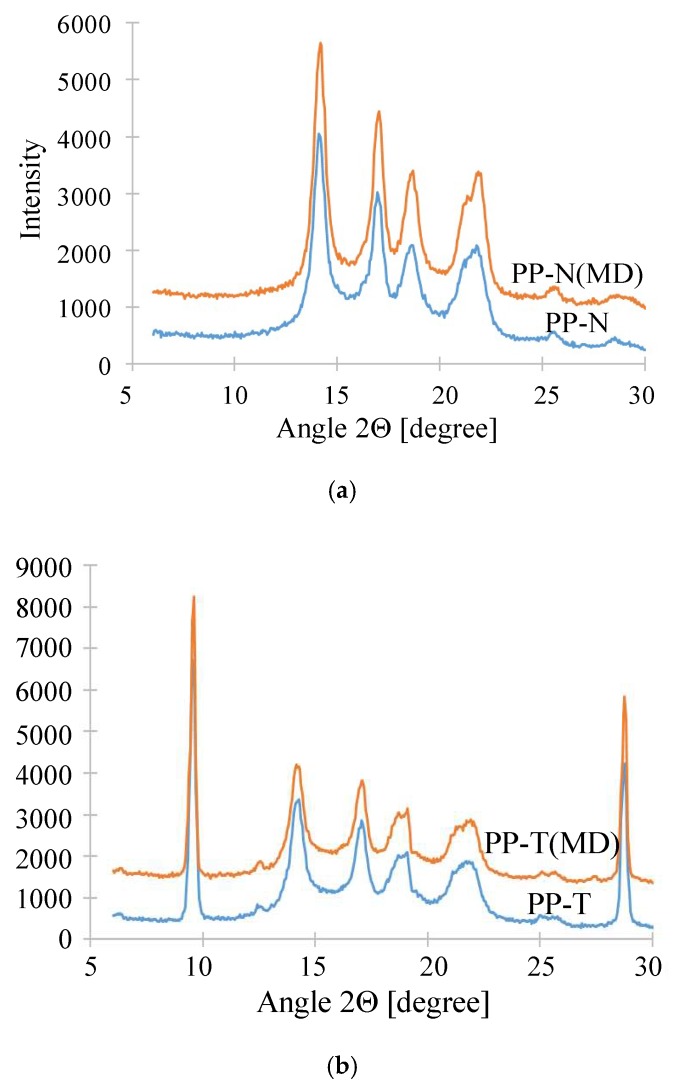
The results of XRD analysis. (**a**) membrane PP-N, (**b**) membrane PP-T.

The differences in composition and crystallinity can be also determined by XRD studies. For the membranes studied, five peaks (14.1, 16.9, 18.5, 21.4 and 21.8°) characteristic of isotactic PP (iPP) were obtained [46]. In the case of PP-T membranes in the XRD spectrum (Figure 10b) there were additional peaks resulting from the presence of talc. These membranes obtained narrower peaks, which confirms that the addition of talc increases the degree of crystallinity of PP membranes (Table 4). Long-term heating of membranes in the MD process promotes recrystallization [62], which is confirmed by changes in full-width at half maximum (FWHM) values determined for membranes after the MD process.

### 3.4. Stability of Membrane Matrix

The membranes for membrane distillation are dry, i.e., the solutions separated by them cannot rinse the components from the membrane matrix. However, in the case of PP membranes after 50–100 h, the pores on the surface of the membranes become wet (partial wetting [50,59]), which allows an elution of components from the membrane matrix. For this reason it is very important that the fillers are permanently embedded in the membranes. The degree of talc leaching from membranes after 350 h of the MD process was evaluated by studying the changes in the concentration of the components forming the membrane matrix. The results obtained for elemental analysis are shown in Table 5.

As can be expected, the membranes blended with talc exhibited higher oxygen content and less carbon. After the MD process, the concentration of all tested components was reduced; hence, the value of the oxygen to carbon ratio was calculated for the assessment of changes. The O/C value for the PP-T membrane decreased from 4.74% to 4.22%, which could indicate a slight loss of talc. However, a similar reduction in the O/C values was also obtained for the PP-N membrane containing no talc. For this reason, on the basis of these data it is difficult to conclude the leaching of talc. In the conducted research, the entire membrane wall was subjected to analysis, including also those areas that were not wetted. This makes it difficult to assess the changes in a thin outer layer of the wall. The sizes of most pore cells were in the range of 1–3 μm; thus, a similar thickness of the rinsed layer can be assumed. At a similar depth, the composition of the membrane wall is analyzed in the SEM-EDS and ATR-FTIR methods. An even thinner layer results in XPS testing.

**Figure 11:**
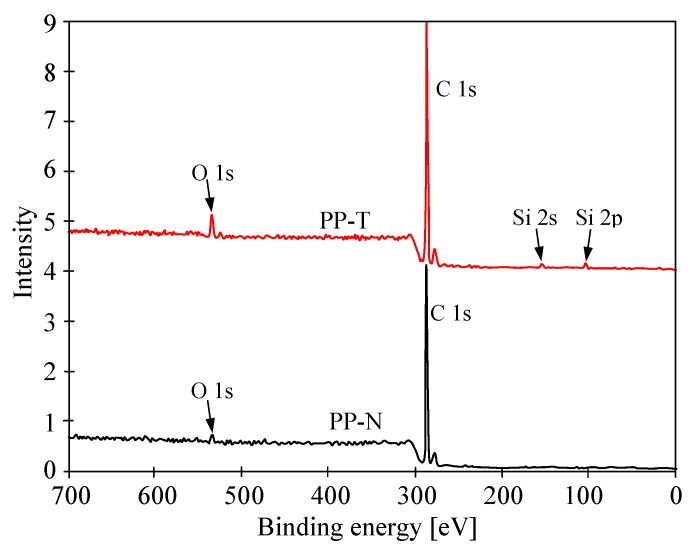
Results of XPS analysis surface of membrane samples (feed side) after 350 h of MD process duration.

The XPS studies analyze the composition of only a few external atomic layers; hence, the results may also include the composition of impurities, in our case Si, which occurs in various materials and could be washed out from the MD installation. However, in the conducted studies, the presence of Si was found only in the case of PP-T membranes (Figure 11). The content of elements detected in the tested membrane samples is shown in Table 6.

The XPS test results can be disturbed by even small amounts of talc that could have deposited on the surface of the membranes during their production. Therefore, the results of tests carried out using the SEM-EDS method for the new membranes are provided (Table 6). It can be concluded that these results are similar to those obtained with the XPS method for membranes after 350 h of the MD process. This confirms that the leaching of talc from the PP-T membrane matrix is practically negligible. Moreover, it can be seen that PP-N membranes did not contain talc, although oxygen was detected in them. The presence of oxygen results from the degradation processes of PP, which results in the formation of e.g., aldehydes and ketones [63].

**Figure 12:**
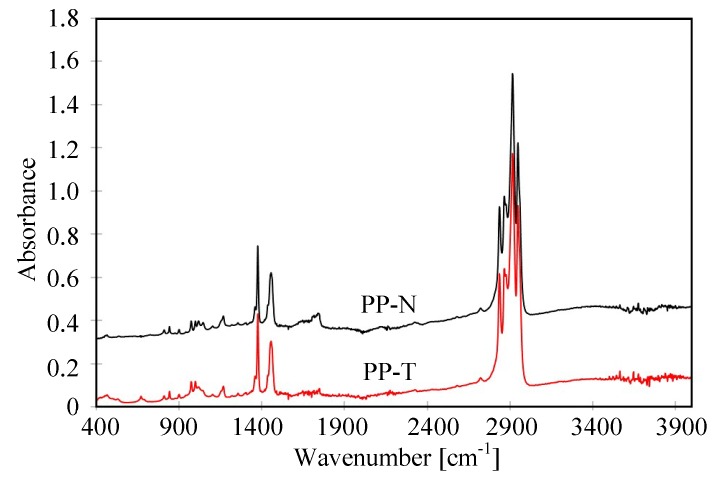
Results of FTIR analysis of tested PP-N and PP-T membranes.

The changes in the membrane surface composition can be tested by the FTIR method. The results obtained for the new membranes PP-N and PP-T are shown in Figure 12. The obtained FTIR spectra have a methyl absorption band at 1375 cm^–1^ and 1450 cm^–1^ characteristic for PP and few peaks (809, 841, 899, 972, 997.1167 and 1219 cm^−1^) attributed to the presence of iPP [64,65]. In the case of PP-N membranes, the significantly larger peaks had an intensity that was observed in the range of 1600–1800 cm^−1^, which is characteristic for the carbonyl groups formed as a result of thermal oxidation [62]. Due to the high temperature of the TIPS process, the polymer degradation already occurs at the membrane production stage [66]. The addition of talc increases the thermal resistance of membranes; hence the degree of degradation of PP-T membranes was reduced. This is also a reason why the PP-T membranes have a larger contact angle (Figure 7).

**Figure 13:**
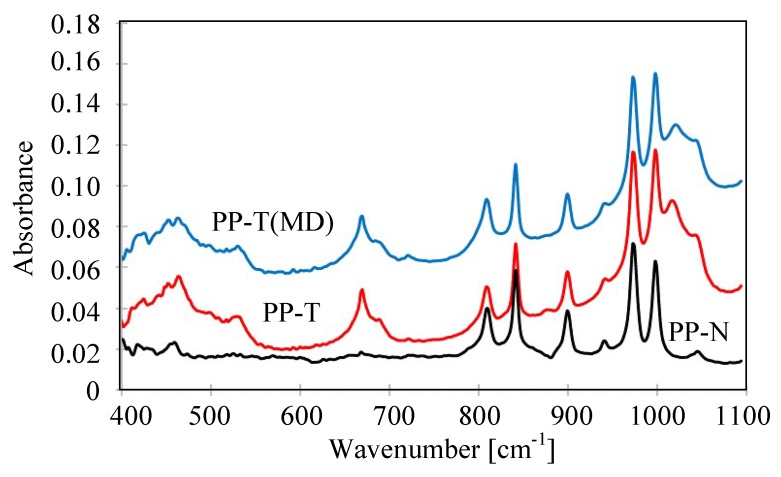
The results of FTIR analysis—peaks characteristic for talc. PP-T(MD) membrane sample colected from MD module after 350 h of the MD process.

The FTIR tests also allows for confirming the presence of talc in the membrane matrix, which show peaks observed in the range of 400–1200 cm^−1^ for the tested membranes (Figure 13). The appearance of the absorbance peak at 1018 cm^−1^ is attributed to the stretching vibrations of Si-O-Si [67]. Large peaks also occur at 660 cm^−1^ and wide in the 400–550 cm^−1^ ranges, which were not observed for the PP-N membrane. A comparison of the data in Figure 13, allows for us to conclude that the intensity of talc peaks is similar for PP-T and PP-T (MD) membranes, confirming the previous results indicating that talc did not rinse during long-term MD process studies.

## 4. Conclusions

The obtained MD results demonstrated that both types of PP membranes used in the work possess the appropriate properties for the MD process, such as a resistance to wetting and almost 100% salt retention. However, the additional tests of the membrane matrix stability revealed that only the PP membranes that were reinforced by talc addition had the properties suitable for application to pilot plant MD studies. Moreover, the MD tests carried out for 350 h did not allow for revealing the occurrence of membrane defects and for this purpose, an even longer MD study should be performed, when the industrial application of membranes is considered.

During the membrane formation via the TIPS method, the flow of melted polypropylene through a spinning nozzle caused a linear arrangement of the polymer chains. A cooling-down of the formed capillary in the coagulation bath proceeded rapidly, favoring the freezing of polymer linear structure. A result is a deterioration of the tensile strength manifested by a longitudinal cracking of polymer. The addition of talc (crystals nucleus) into a dope solution caused the disturbance in the linear arrangement of the polymer chains, and PP membranes with a surface morphology more akin to a sponge-like structure was formed. In this case, the MD process efficiency was higher by more than 10% in comparison to the utilization of membranes without talc addition.

In the MD process, the membranes are in contact with the feed at a high temperature, e.g., 353 K, which causes the formation of thermal stress resulting in the numerous cracks which were observed on the surface of membranes having a linear structure. Such problems were not found in the case of membranes where the linear arrangement of polymer chains was disturbed by the addition of talc into a dope solution.

It has been confirmed that the uniform dispersion of talc was achieved within the membrane formed by the TIPS method. Talc was permanently incorporated into the polypropylene matrix and did not leach from the PP membranes during the MD process.

## Figures and Tables

**Table 1:** The parameters of applied membranes.

Membrane	Internal Diameter [mm]	Wall [mm]	Porosity [%]	Pore Diameter [μm]
PP-N	1.8	0.4	81	0.2
PP-T	1.8	0.4	84	0.2

**Table 2:** The mechanical properties of studied PP membranes.

Membrane	Young’s Modulus [MPa]	Elongation at Break [%]	Tensile Strength [MPa]
PP-N	106.1 +/− 18.3	154.5 +/− 26.8	1.98 +/− 0.13
PP-T	135.5 +/− 12.8	172.2 +/− 19.7	2.55 +/− 0.14

**Table 3:** The results of differential scanning calorimetry (DSC) analysis of new membranes and membrane samples after MD studies.

Membrane	T_m_ [°C]	ΔH_m_ [J/g]	T_C_ [°C]	ΔH_C_ [J/g]	X [%]
PP-N	164.6	91.2	116.1	82.3	44
PP-N (MD)	157.2	84.9	117.1	68.4	41
PP-T	163.7	117.1	119.1	93.2	56
PP-T (MD)	162.7	109.8	114.6	91.7	53

**Table 4:** The values of full-width at half maximum (FWHM) of the peaks obtained during XRD analysis.

Membrane	Peak 14.1 [°]	Peak 16.9 [°]
PP-N	0.89 +/− 0.01	1.0 +/− 0.02
PP-N (MD)	0.72 +/− 0.01	0.74 +/− 0.02
PP-T	0.67 +/− 0.01	0.71 +/− 0.02
PP-T (MD)	0.69 +/− 0.01	0.68 +/− 0.01

**Table 5:** The results of elemental analysis.

Membrane	C [%]	O [%]	H [%]	O/C [%]
PP-N	85.613 +/− 0.811	0.976 +/− 0.009	14.423 +/− 0.126	1.14
PP-N (MD)	82.668 +/− 0.116	0.804 +/− 0.116	13.961 +/− 0.116	0.97
PP-T	79.788 +/− 0.116	3.783 +/− 0.281	13.312 +/− 0.015	4.74
PP-T (MD)	76.333 +/− 0.116	3.224 +/− 0.116	12.856 +/− 0.116	4.22

**Table 6:** The results of EDS and XPS analysis of membrane samples.

Membrane	C [%]	O [%]	Si [%]	Mg [%]
PP-N (EDS)	98.3	0.9	-	-
PP-N (XPS)	98.1	1.9	-	-
PP-T (EDS)	93.1	2.7	2.2	1.8
PP-T (XPS)	91.8	4.2	2.5	1.4
